# Supplementary material for: Spatial metabolomics for symbiotic marine invertebrates
Source: Life Sci Alliance. 2023 May 18;6(8):e202301900. doi: 10.26508/lsa.202301900 (PMC10200813; doi:10.26508/lsa.202301900)
Supplement: Supplementary file 2 [file LSA-2023-01900_TableS1.docx]

**Table S1. Full names of metabolite abbreviations of the major groups.**

| **Abbreviation** | **Full name** | **Category** |
| --- | --- | --- |
| CAR | Acyl carnitine | Energy homeostasis |
| Cer | Ceramide | Signaling |
| Chl F | Chlorophyll fragment | Energy/photosynthesis |
| DG | Diglyceride | Energy |
| DGCC | Diacylglycerylcarboxyhydroxymethylcholine | Structural |
| DGDG | Digalactosyldiacylglycerol | Structural |
| DGTA | Diacylglycerylhydroxymethyltrimethylalanine | Structural |
| DGTS | Diacylglyceryltrimethylhomoserine | Structural |
| FA | Fatty acids/esters | Energy/backbone |
| FA CONJ | Fatty acid conjugation | Energy/signaling |
| GlcCer | Glucosylceramide | Signaling/immune/cellular recognition |
| HexCer | Hexosylceramide | Signaling/immune/cellular recognition |
| HG | Headgroup | Structural |
| LPA | Lysophosphatidic acid | Signaling |
| LPC | Lysophosphatidylcholine | Structural |
| LPI | Lysophosphatidylinositol | Structural |
| MG | Mono(acyl/alkyl)glycerol | Energy |
| MGCC | Monoacylglycerylcarboxyhydroxymethylcholine | Structural |
| MIPC | Mannosylinositol phosphorylceramide | Signaling/immune/cellular recognition |
| PA | Phosphatidic acid | Signaling |
| PC | Glycerophosphocholine | Structural |
| PE | Phosphatidylethanolamine | Structural |
| PG | Phosphoglycan | Structural/immune |
| Pheo a | Pheophorbide a | Energy/photosynthesis |
| PI | Phosphatidylinositol | Structural/signaling |
| PIP | Phosphatidylinositol-*n*-phosphate | Structural/signaling |
| PM | Polar metabolite | All |
| PS | Phosphatidylserine | Structural/signaling |
| SQDG | Sulfoquinovosyl diacylglycerol | Structural/energy/photosynthesis |
| ST | Sterol | Structural/signaling |
| TG | Triglyceride | Energy |
