# Supplementary material for: Spatial metabolomics for symbiotic marine invertebrates
Source: Life Sci Alliance. 2023 May 18;6(8):e202301900. doi: 10.26508/lsa.202301900 (PMC10200813; doi:10.26508/lsa.202301900)
Supplement: Supplementary file 3 [file LSA-2023-01900_TableS2.docx]

**Table S2. Ceramides with significantly different relative intensity between C1-anemones and B1-anemones.**

| **m/z** | **t.stat** | **P_adj_** | **FC*** | **log2(FC)** |
| --- | --- | --- | --- | --- |
| 558.487_Cer/GlcCer | -6.8 | 0.005 | 0.09 | -3.51 |
| 598.504_Cer | 9.8 | 0.002 | 14.27 | 3.84 |
| 624.520_Cer | 5.0 | 0.01 | 6.18 | 2.63 |
| 638.535_Cer/CAR | 11.4 | 0.002 | 13.30 | 3.73 |
| 640.551_Cer/CAR/DGTS | 5.1 | 0.01 | 5.35 | 2.42 |
| 650.536_Cer/CAR | 16.1 | 0.001 | 10.91 | 3.45 |
| 652.551_Cer/CAR | 5.3 | 0.01 | 6.42 | 2.68 |
| 653.555_Cer/CAR | 12.8 | 0.002 | 13.48 | 3.75 |
| 666.567_Cer/CAR/DG | 10.7 | 0.002 | 11.72 | 3.55 |
| 668.582_Cer/CAR | 8.5 | 0.003 | 4.34 | 2.12 |
| 676.552_Cer/CAR/LPC | 8.0 | 0.003 | 11.17 | 3.48 |
| 680.583_Cer/CAR/DGTS | 9.1 | 0.002 | 10.69 | 3.42 |
| 682.597_Cer/CAR | 5.1 | 0.01 | 7.25 | 2.86 |
| 708.613_Cer | 7.7 | 0.003 | 20.74 | 4.37 |
| 714.591_HexCer/DGCC | -4.3 | 0.017 | 0.17 | -2.58 |
| 716.607_HexCer/Cer | -3.8 | 0.023 | 0.27 | -1.90 |
| 730.622_HexCer | -5.6 | 0.009 | 0.19 | -2.43 |
| 994.575_MIPC/Hex2Cer | -9.2 | 0.002 | 0.18 | -2.47 |
| 1050.70_MIPC/Hex2Cer | -8.0 | 0.003 | 0.05 | -4.39 |
| 1094.69_MIPC/Hex2Cer | -45.4 | <0.001 | 0.03 | -5.21 |
| 1108.71_MIPC/Hex2Cer | -9.4 | 0.002 | 0.05 | -4.39 |

*A fold change (FC) > 1 suggests that this ceramide had higher relative intensity in B1-anemones, whereas a FC < 1 indicates that this metabolite had higher relative intensity in C1-anemones. For instance, a FC value of 0.1 means that this ceramide had 10 times high relative intensity in C1-anemones than B1-anemones.
