# Supplementary material for: Spatial metabolomics for symbiotic marine invertebrates
Source: Life Sci Alliance. 2023 May 18;6(8):e202301900. doi: 10.26508/lsa.202301900 (PMC10200813; doi:10.26508/lsa.202301900)
Supplement: Supplementary file 4 [file LSA-2023-01900_TableS3.docx]

**Table S3. ANOVA and Tukey's HSD results of betaine lipids.** Anemone groups for comparisons were B1-anemones (B1), C1-anemones (C1) and aposymbiotic anemones.

| **m/z** | **f.value** | **FDR** | **Significant pairs** |
| --- | --- | --- | --- |
| 472.363_DGTS | 60.4 | <0.001 | B1-Aposymbiotic; C1-Aposymbiotic |
| 490.374_MGCC | 229.4 | <0.001 | B1-Aposymbiotic; C1-Aposymbiotic |
| 512.359_MGCC | 18.9 | 0.004 | B1-Aposymbiotic; C1-Aposymbiotic |
| 562.374_MGCC | 87.0 | <0.001 | B1-Aposymbiotic; C1-Aposymbiotic |
| 763.512_DGCC | 80.4 | <0.001 | B1-Aposymbiotic; C1-Aposymbiotic; C1-B1 |
| 772.572_DGCC | 43.5 | <0.001 | B1-Aposymbiotic; C1-Aposymbiotic; C1-B1 |
| 774.589_DGCC | 61.7 | <0.001 | C1-Aposymbiotic; C1-B1 |
| 798.587_DGCC | 16.5 | 0.005 | B1-Aposymbiotic; C1-Aposymbiotic |
| 800.604_DGCC | 228.5 | <0.001 | B1-Aposymbiotic; C1-Aposymbiotic |
| 822.586_DGCC | 10.5 | 0.013 | B1-Aposymbiotic; C1-Aposymbiotic |
| 823.588_DGCC | 14.1 | 0.007 | B1-Aposymbiotic; C1-Aposymbiotic |
