# Supplementary material for: Spatial metabolomics for symbiotic marine invertebrates
Source: Life Sci Alliance. 2023 May 18;6(8):e202301900. doi: 10.26508/lsa.202301900 (PMC10200813; doi:10.26508/lsa.202301900)
Supplement: Supplementary file 5 [file LSA-2023-01900_TableS4.docx]

**Table S4. T-test results and fold changes of metabolites with significantly different relative intensity between aposymbiotic and symbiotic anemones.**

| **m/z** | **t.stat** | **P_adj_** | **FC*** | **log2(FC)** |
| --- | --- | --- | --- | --- |
| 383.288_FA/ST | 3.0 | 0.050 | 11.93 | 3.58 |
| 397.303_FA | 3.5 | 0.032 | 27.45 | 4.78 |
| 465.353_FA CONJ | -4.0 | 0.020 | 0.02 | -5.39 |
| 472.363_DGTS | -9.1 | 0.001 | 0.02 | -5.97 |
| 490.374_MGCC | -15.8 | <0.001 | 0.002 | -9.32 |
| 512.359_MGCC | -5.3 | 0.006 | 0.04 | -4.72 |
| 532.509_Cer | 6.8 | 0.002 | 38.07 | 5.25 |
| 562.374_MGCC | -14.0 | <0.001 | 0.002 | -8.72 |
| 592.268_Pheo a | -3.3 | 0.039 | 0.07 | -3.77 |
| 593.276_Pheo a | -3.4 | 0.035 | 0.05 | -4.24 |
| 614.427_Chl F | -3.7 | 0.027 | 0.06 | -3.94 |
| 625.507_DG | 5.1 | 0.007 | 22.41 | 4.49 |
| 626.536_Cer | -7.6 | 0.002 | 0.02 | -5.44 |
| 641.352_PM | 3.1 | 0.045 | 5.60 | 2.49 |
| 654.567_Cer/CAR | -6.8 | 0.002 | 0.04 | -4.54 |
| 655.570_DG | -5.2 | 0.007 | 0.03 | -5.16 |
| 661.515_LPA | 3.5 | 0.030 | 22.78 | 4.51 |
| 682.597_Cer/CAR | -4.7 | 0.010 | 0.02 | -5.88 |
| 693.373_PG/LPG | -3.8 | 0.024 | 0.12 | -3.07 |
| 714.591_HexCer/DGCC | -4.1 | 0.017 | 0.03 | -5.01 |
| 717.577_DG | 3.8 | 0.024 | 27.77 | 4.80 |
| 732.503_PC | -3.8 | 0.024 | 0.12 | -3.03 |
| 732.554_PC/PE | 5.5 | 0.005 | 9.19 | 3.20 |
| 733.516_PA | 3.4 | 0.033 | 7.68 | 2.94 |
| 734.556_DGCC | 3.2 | 0.044 | 11.13 | 3.48 |
| 740.559_PC | 8.9 | 0.001 | 15.05 | 3.91 |
| 741.561_DG/TG | 3.1 | 0.045 | 6.43 | 2.69 |
| 744.590_PC | 9.8 | 0.001 | 7.83 | 2.97 |
| 745.479_PG | 3.1 | 0.048 | 6.51 | 2.70 |
| 746.461_DGCC | -3.4 | 0.035 | 0.09 | -3.49 |
| 746.605_PC/PE | 3.7 | 0.027 | 3.55 | 1.83 |
| 747.609_DG/TG | 4.4 | 0.013 | 4.29 | 2.10 |
| 748.572_DGCC | -3.4 | 0.033 | 0.04 | -4.70 |
| 749.576_DG/TG | -3.5 | 0.030 | 0.05 | -4.36 |
| 756.590_PC | 5.1 | 0.007 | 25.22 | 4.66 |
| 759.573_PC/PE | 8.2 | 0.001 | 7.49 | 2.90 |
| 760.534_PC/PE | -4.4 | 0.013 | 0.15 | -2.78 |
| 760.585_PC/PE | 5.3 | 0.006 | 5.63 | 2.49 |
| 761.588_PC/PE | 5.0 | 0.008 | 6.28 | 2.65 |
| 763.512_DGCC | -8.2 | 0.001 | 0.005 | -7.70 |
| 764.558_PC | 4.3 | 0.014 | 3.92 | 1.97 |
| 765.560_PC/PE | 4.2 | 0.015 | 6.13 | 2.62 |
| 768.591_PC | 3.1 | 0.045 | 2.33 | 1.22 |
| 769.593_PC | 3.7 | 0.027 | 2.58 | 1.37 |
| 770.605_PC | 11.2 | <0.001 | 27.02 | 4.76 |
| 772.572_DGCC | -4.7 | 0.010 | 0.03 | -5.21 |
| 773.171_PM | -3.1 | 0.047 | 0.01 | -7.17 |
| 779.486_PG | -6.4 | 0.003 | 0.02 | -5.38 |
| 782.568_PC | 10.8 | <0.001 | 11.62 | 3.54 |
| 784.534_PC | -5.0 | 0.008 | 0.11 | -3.21 |
| 784.584_PC | 9.8 | 0.001 | 22.26 | 4.48 |
| 785.453_SQDG | -7.1 | 0.002 | 0.03 | -4.98 |
| 787.604_PC | 8.8 | 0.001 | 43.15 | 5.43 |
| 789.620_PA/TG | 5.7 | 0.005 | 26.94 | 4.75 |
| 796.622_PC | 3.3 | 0.036 | 3.23 | 1.69 |
| 798.587_DGCC | -6.2 | 0.003 | 0.05 | -4.20 |
| 800.604_DGCC | -22.8 | <0.001 | 0.05 | -9.35 |
| 802.536_PC | 3.5 | 0.030 | 7.29 | 2.87 |
| 806.567_PC | 5.9 | 0.004 | 5.27 | 2.40 |
| 807.571_PC | 6.7 | 0.002 | 6.35 | 2.67 |
| 808.584_PC | 7.1 | 0.002 | 8.37 | 3.07 |
| 809.587_PC | 7.7 | 0.002 | 11.57 | 3.53 |
| 810.600_PC | 5.6 | 0.005 | 7.62 | 2.93 |
| 811.604_PC | 5.4 | 0.006 | 10.95 | 3.45 |
| 822.586_DGCC | -4.4 | 0.013 | 0.07 | -3.93 |
| 822.636_PC | 5.4 | 0.006 | 39.27 | 5.30 |
| 823.588_DGCC | -4.8 | 0.009 | 0.04 | -4.73 |
| 834.601_PC | 3.5 | 0.030 | 6.23 | 2.64 |
| 835.603_PC | 3.4 | 0.035 | 8.40 | 3.07 |
| 836.615_PC | 4.9 | 0.008 | 13.13 | 3.71 |
| 838.633_PC | 3.9 | 0.020 | 6.37 | 2.67 |
| 839.563_Chl | -6.6 | 0.003 | 0.04 | -4.74 |
| 839.635_PC | 4.1 | 0.018 | 7.45 | 2.90 |
| 870.564_PC | -6.5 | 0.003 | 0.02 | -5.84 |
| 988.686_PC | -3.2 | 0.040 | 0.05 | -4.42 |
| 1019.61_PIP/DGDG | 3.4 | 0.033 | 5.48 | 2.45 |
| 400.302 | -12.1 | <0.001 | 0.01 | -6.43 |
| 400.804 | -6.9 | 0.002 | 0.03 | -5.25 |
| 464.35 | -4.8 | 0.009 | 0.09 | -3.44 |
| 491.377 | -26.1 | <0.001 | 0.01 | -6.87 |
| 496.376 | 6.5 | 0.003 | 7.22 | 2.85 |
| 497.38 | 5.3 | 0.006 | 9.16 | 3.20 |
| 500.483 | 7.5 | 0.002 | 23.38 | 4.55 |
| 508.377 | 5.1 | 0.007 | 36.62 | 5.19 |
| 518.493 | 5.8 | 0.005 | 8.91 | 3.16 |
| 519.497 | 5.2 | 0.007 | 16.64 | 4.06 |
| 522.355 | 4.7 | 0.010 | 15.68 | 3.97 |
| 524.145 | 4.0 | 0.020 | 24.29 | 4.60 |
| 524.371 | 6.9 | 0.002 | 9.71 | 3.28 |
| 524.407 | 6.1 | 0.004 | 6.53 | 2.71 |
| 525.374 | 4.5 | 0.011 | 14.46 | 3.85 |
| 547.357 | 5.1 | 0.007 | 8.59 | 3.10 |
| 562.158 | 4.5 | 0.012 | 12.50 | 3.64 |
| 563.378 | -11.3 | <0.001 | 0.003 | -8.35 |
| 564.381 | -3.7 | 0.025 | 0.06 | -3.99 |
| 568.135 | 4.1 | 0.018 | 28.37 | 4.83 |
| 578.369 | -3.8 | 0.023 | 0.05 | -4.40 |
| 581.399 | -8.4 | 0.001 | 0.01 | -6.74 |
| 582.402 | -5.3 | 0.006 | 0.02 | -5.32 |
| 584.356 | -3.7 | 0.027 | 0.04 | -4.75 |
| 590.322 | -4.5 | 0.012 | 0.01 | -6.14 |
| 592.035 | 3.4 | 0.033 | 7.46 | 2.90 |
| 602.427 | -4.5 | 0.012 | 0.04 | -4.56 |
| 606.296 | -4.0 | 0.020 | 0.01 | -6.30 |
| 612.519 | -3.3 | 0.037 | 0.05 | -4.47 |
| 621.421 | -3.1 | 0.048 | 0.22 | -2.19 |
| 622.026 | -7.4 | 0.002 | 0.02 | -5.62 |
| 622.084 | -3.6 | 0.027 | 0.05 | -4.24 |
| 626.427 | -5.1 | 0.007 | 0.05 | -4.22 |
| 636.223 | -3.4 | 0.035 | 0.03 | -5.15 |
| 643.517 | 4.0 | 0.019 | 6.07 | 2.60 |
| 644.453 | -3.6 | 0.028 | 0.03 | -5.11 |
| 662.518 | 3.9 | 0.021 | 20.74 | 4.37 |
| 665.5 | 7.1 | 0.002 | 3.51 | 1.81 |
| 666.504 | 5.1 | 0.007 | 4.05 | 2.02 |
| 667.506 | 5.0 | 0.008 | 9.79 | 3.29 |
| 674.163 | -13.6 | <0.001 | 0.02 | -5.66 |
| 679.457 | -3.8 | 0.024 | 0.30 | -1.74 |
| 680.519 | 6.8 | 0.002 | 16.08 | 4.01 |
| 742.575 | 12.8 | <0.001 | 7.77 | 2.96 |
| 743.578 | 11.3 | <0.001 | 9.21 | 3.20 |
| 744.493 | -3.4 | 0.034 | 0.22 | -2.21 |
| 745.495 | -4.7 | 0.010 | 0.12 | -3.00 |
| 745.593 | 10.6 | <0.001 | 10.78 | 3.43 |
| 748.477 | -3.5 | 0.033 | 0.14 | -2.86 |
| 748.612 | 3.0 | 0.050 | 5.23 | 2.39 |
| 751.592 | -3.2 | 0.040 | 0.15 | -2.73 |
| 752.603 | -8.8 | 0.001 | 0.05 | -4.31 |
| 754.536 | 10.5 | <0.001 | 28.06 | 4.81 |
| 754.619 | -12.8 | <0.001 | 0.02 | -6.05 |
| 755.498 | -15.5 | <0.001 | 0.02 | -5.91 |
| 755.623 | -6.0 | 0.004 | 0.05 | -4.42 |
| 756.503 | -10.3 | <0.001 | 0.01 | -6.65 |
| 756.554 | 8.6 | 0.001 | 5.95 | 2.57 |
| 757.557 | 7.6 | 0.002 | 10.84 | 3.44 |
| 758.569 | 5.2 | 0.007 | 6.03 | 2.59 |
| 760.622 | 6.7 | 0.003 | 11.19 | 3.48 |
| 762.59 | 3.8 | 0.024 | 8.02 | 3.00 |
| 764.516 | -5.8 | 0.004 | 0.02 | -5.97 |
| 768.577 | 3.1 | 0.047 | 2.53 | 1.34 |
| 769.478 | -5.9 | 0.004 | 0.05 | -4.38 |
| 770.482 | -6.1 | 0.004 | 0.04 | -4.57 |
| 771.473 | -12.4 | <0.001 | 0.01 | -6.88 |
| 771.609 | 9.7 | 0.001 | 30.03 | 4.91 |
| 772.477 | -13.4 | <0.001 | 0.02 | -5.93 |
| 772.525 | 4.5 | 0.012 | 4.42 | 2.14 |
| 772.621 | 10.7 | <0.001 | 23.38 | 4.55 |
| 773.575 | -3.7 | 0.026 | 0.06 | -3.95 |
| 773.624 | 8.8 | 0.001 | 63.33 | 5.98 |
| 774.601 | 4.3 | 0.014 | 12.10 | 3.60 |
| 774.636 | 4.2 | 0.015 | 10.75 | 3.43 |
| 775.64 | 6.8 | 0.002 | 10.81 | 3.43 |
| 778.537 | 3.1 | 0.046 | 2.80 | 1.49 |
| 779.363 | -3.0 | 0.048 | 0.05 | -4.45 |
| 780.552 | 7.2 | 0.002 | 5.25 | 2.39 |
| 780.589 | 3.6 | 0.027 | 5.16 | 2.37 |
| 781.556 | 5.8 | 0.005 | 8.73 | 3.13 |
| 781.593 | 3.9 | 0.020 | 7.47 | 2.90 |
| 783.53 | -7.5 | 0.002 | 0.05 | -4.37 |
| 783.571 | 11.0 | <0.001 | 11.18 | 3.48 |
| 785.588 | 8.0 | 0.001 | 27.49 | 4.78 |
| 786.601 | 9.7 | 0.001 | 21.02 | 4.39 |
| 788.615 | 6.1 | 0.004 | 17.09 | 4.09 |
| 794.607 | 5.4 | 0.006 | 5.54 | 2.47 |
| 795.608 | 4.9 | 0.009 | 7.01 | 2.81 |
| 797.624 | 3.3 | 0.037 | 3.43 | 1.78 |
| 798.628 | 8.9 | 0.001 | 11.01 | 3.46 |
| 801.606 | -16.3 | <0.001 | 0.001 | -9.78 |
| 802.611 | -14.9 | <0.001 | 0.002 | -8.83 |
| 803.613 | -10.1 | <0.001 | 0.02 | -5.94 |
| 804.552 | 6.3 | 0.003 | 11.66 | 3.54 |
| 805.554 | 5.1 | 0.007 | 15.02 | 3.91 |
| 808.562 | 3.8 | 0.024 | 12.97 | 3.70 |
| 808.621 | 4.1 | 0.018 | 8.26 | 3.05 |
| 810.451 | -5.6 | 0.005 | 0.02 | -5.82 |
| 810.464 | -3.2 | 0.040 | 0.17 | -2.58 |
| 812.616 | 8.5 | 0.001 | 58.00 | 5.86 |
| 814.571 | -10.3 | <0.001 | 0.13 | -2.96 |
| 815.575 | -10.5 | <0.001 | 0.07 | -3.88 |
| 815.622 | -4.6 | 0.011 | 0.01 | -6.29 |
| 816.588 | 3.6 | 0.028 | 2.73 | 1.45 |
| 816.597 | -8.2 | 0.001 | 0.01 | -6.10 |
| 817.592 | 3.1 | 0.046 | 2.93 | 1.55 |
| 820.526 | 3.2 | 0.044 | 13.47 | 3.75 |
| 822.541 | 3.0 | 0.050 | 9.90 | 3.31 |
| 828.551 | -4.5 | 0.012 | 0.28 | -1.85 |
| 828.635 | -10.5 | <0.001 | 0.02 | -5.86 |
| 829.556 | -4.8 | 0.009 | 0.24 | -2.04 |
| 830.546 | -4.2 | 0.016 | 0.08 | -3.59 |
| 831.549 | -7.5 | 0.002 | 0.03 | -5.24 |
| 832.583 | 5.6 | 0.005 | 4.89 | 2.29 |
| 832.593 | -6.3 | 0.003 | 0.03 | -5.22 |
| 833.587 | 5.7 | 0.005 | 6.12 | 2.61 |
| 838.559 | -5.0 | 0.008 | 0.12 | -3.04 |
| 842.604 | -3.1 | 0.046 | 0.36 | -1.47 |
| 843.607 | -4.6 | 0.011 | 0.19 | -2.43 |
| 845.529 | -3.6 | 0.028 | 0.13 | -2.97 |
| 854.235 | -3.1 | 0.045 | 0.01 | -7.31 |
| 854.244 | -3.3 | 0.037 | 0.01 | -7.60 |
| 854.543 | 5.1 | 0.007 | 8.09 | 3.02 |
| 859.579 | -3.1 | 0.047 | 0.15 | -2.71 |
| 864.585 | -5.7 | 0.005 | 0.03 | -5.28 |
| 866.681 | -5.3 | 0.007 | 0.05 | -4.37 |
| 867.683 | -5.2 | 0.007 | 0.03 | -5.25 |
| 871.571 | -6.5 | 0.003 | 0.01 | -7.63 |
| 872.577 | -5.0 | 0.008 | 0.02 | -5.97 |
| 872.603 | -7.0 | 0.002 | 0.02 | -5.98 |
| 873.607 | -5.2 | 0.007 | 0.03 | -5.03 |
| 878.816 | -3.1 | 0.045 | 0.04 | -4.59 |
| 880.833 | -3.4 | 0.035 | 0.02 | -6.02 |
| 885.646 | -6.5 | 0.003 | 0.04 | -4.81 |
| 893.503 | -3.3 | 0.036 | 0.24 | -2.05 |
| 893.71 | -3.7 | 0.025 | 0.18 | -2.48 |
| 899.406 | -3.3 | 0.037 | 0.18 | -2.51 |
| 906.847 | -3.5 | 0.030 | 0.02 | -5.68 |
| 907.85 | -3.6 | 0.027 | 0.03 | -5.09 |
| 910.714 | -12.6 | <0.001 | 0.005 | -7.69 |
| 911.716 | -8.7 | 0.001 | 0.01 | -6.53 |
| 912.72 | -6.1 | 0.004 | 0.03 | -5.30 |
| 920.385 | -4.1 | 0.018 | 0.09 | -3.49 |
| 930.849 | -3.1 | 0.045 | 0.03 | -5.23 |
| 932.866 | -4.4 | 0.013 | 0.03 | -5.26 |
| 954.603 | 3.4 | 0.035 | 5.91 | 2.56 |
| 981.63 | 6.6 | 0.003 | 9.98 | 3.32 |
| 982.634 | 7.4 | 0.002 | 13.28 | 3.73 |
| 983.646 | 10.1 | <0.001 | 20.38 | 4.35 |
| 985.662 | 4.6 | 0.011 | 5.61 | 2.49 |
| 989.644 | -4.3 | 0.014 | 0.05 | -4.27 |
| 993.592 | 4.3 | 0.014 | 10.99 | 3.46 |
| 994.598 | 4.6 | 0.011 | 12.74 | 3.67 |
| 995.61 | 5.6 | 0.005 | 10.29 | 3.36 |
| 996.613 | 5.2 | 0.007 | 12.35 | 3.63 |
| 997.627 | 6.3 | 0.003 | 16.26 | 4.02 |
| 999.642 | 4.9 | 0.008 | 10.47 | 3.39 |
| 1003.61 | -9.6 | 0.001 | 0.22 | -2.19 |
| 1004.62 | -7.1 | 0.002 | 0.18 | -2.50 |
| 1005.62 | -5.3 | 0.006 | 0.11 | -3.17 |
| 1005.63 | 3.8 | 0.024 | 3.88 | 1.96 |
| 1019.59 | -5.6 | 0.005 | 0.07 | -3.76 |
| 1020.59 | -6.2 | 0.003 | 0.05 | -4.34 |
| 1021.62 | 3.3 | 0.037 | 5.55 | 2.47 |
| 1022.63 | 3.7 | 0.025 | 6.48 | 2.70 |
| 1043.58 | 5.5 | 0.005 | 14.31 | 3.84 |
| 1044.59 | 5.8 | 0.005 | 15.95 | 4.00 |
| 1054.72 | -4.7 | 0.010 | 0.12 | -3.02 |
| 1055.72 | -3.5 | 0.032 | 0.17 | -2.57 |
| 1146.68 | 6.9 | 0.002 | 7.29 | 2.87 |
| 1160.65 | 3.4 | 0.035 | 5.69 | 2.51 |
| 1215.63 | -3.0 | 0.050 | 0.09 | -3.50 |

*A fold change (FC) > 1 suggests that this metabolite had higher relative intensity in aposymbiotic anemones, whereas a FC < 1 indicates that this metabolite had higher relative intensity in symbiotic anemones. For instance, a FC value of 0.1 means that this metabolite had 10 times high relative intensity in symbiotic than aposymbiotic anemones.
