# Supplementary material for: Spatial metabolomics for symbiotic marine invertebrates
Source: Life Sci Alliance. 2023 May 18;6(8):e202301900. doi: 10.26508/lsa.202301900 (PMC10200813; doi:10.26508/lsa.202301900)
Supplement: Supplementary file 6 [file LSA-2023-01900_TableS5.docx]

**Table S5. T-test results and fold changes of metabolites with significantly different relative intensity between B1-anemones and C1-anemones.**

| **m/z** | **t.stat** | **P_adj_** | **FC*** | **log2(FC)** |
| --- | --- | --- | --- | --- |
| 184.073_PC HG | 9.6 | 0.012 | 2839.70 | 11.47 |
| 241.180_FA | 6.9 | 0.017 | 113.10 | 6.82 |
| 256.203_FA | 5.6 | 0.027 | 116.01 | 6.86 |
| 264.204_FA/MG | 8.5 | 0.014 | 126.40 | 6.98 |
| 336.225_CAR | -9.5 | 0.012 | 0.002 | -9.02 |
| 337.229_CAR | -18.7 | 0.012 | 0.001 | -9.49 |
| 358.304_CAR/FA | -8.3 | 0.014 | 0.04 | -4.52 |
| 397.303_FA | 5.9 | 0.025 | 47.79 | 5.58 |
| 402.198_FA CONJ | -11.6 | 0.012 | 0.01 | -6.60 |
| 452.181_FA CONJ | -9.7 | 0.012 | 0.06 | -4.17 |
| 465.353_FA CONJ | -4.3 | 0.047 | 0.12 | -3.02 |
| 482.284_Chl F | -5.7 | 0.027 | 0.05 | -4.25 |
| 526.274_Lyso-lipid | -7.1 | 0.017 | 0.04 | -4.78 |
| 545.239_ST | -5.3 | 0.029 | 0.05 | -4.42 |
| 558.487_Cer/GlcCer | -6.8 | 0.018 | 0.09 | -3.51 |
| 573.488_WE/DG | -6.5 | 0.020 | 0.08 | -3.70 |
| 575.226_LPC | -6.8 | 0.018 | 0.03 | -5.13 |
| 597.235_LPI | -9.8 | 0.012 | 0.02 | -5.62 |
| 598.504_Cer | 9.8 | 0.012 | 14.27 | 3.84 |
| 609.524_DG | -5.3 | 0.030 | 0.18 | -2.48 |
| 624.520_Cer | 5.0 | 0.033 | 6.18 | 2.63 |
| 635.233_PM | -5.0 | 0.034 | 0.22 | -2.17 |
| 636.236_PM | -11.9 | 0.012 | 0.12 | -3.11 |
| 638.535_Cer/CAR | 11.4 | 0.012 | 13.30 | 3.73 |
| 640.551_Cer/CAR/DGTS | 5.1 | 0.032 | 5.35 | 2.42 |
| 641.225_PM | -7.5 | 0.017 | 0.04 | -4.75 |
| 641.555_DG | 7.9 | 0.015 | 10.44 | 3.38 |
| 650.536_Cer/CAR | 16.1 | 0.012 | 10.91 | 3.45 |
| 652.551_Cer/CAR | 5.3 | 0.030 | 6.42 | 2.68 |
| 653.555_Cer/CAR | 12.8 | 0.012 | 13.48 | 3.75 |
| 655.570_DG | 4.6 | 0.040 | 4.84 | 2.27 |
| 656.573_DG | 8.6 | 0.014 | 8.88 | 3.15 |
| 657.216_PM | -10.6 | 0.012 | 0.26 | -1.94 |
| 666.567_Cer/CAR/DG | 10.7 | 0.012 | 11.72 | 3.55 |
| 668.582_Cer/CAR | 8.5 | 0.014 | 4.34 | 2.12 |
| 676.552_Cer/CAR/LPC | 8.0 | 0.015 | 11.17 | 3.48 |
| 680.183_PM | -4.8 | 0.036 | 0.06 | -4.10 |
| 680.583_Cer/CAR/DGTS | 9.1 | 0.012 | 10.69 | 3.42 |
| 681.585_DG | 12.5 | 0.012 | 23.93 | 4.58 |
| 682.597_Cer/CAR | 5.1 | 0.032 | 7.25 | 2.86 |
| 693.373_PG/LPG | -5.7 | 0.026 | 0.26 | -1.95 |
| 705.484_PA | -5.9 | 0.025 | 0.15 | -2.76 |
| 706.487_PA | -5.6 | 0.027 | 0.11 | -3.19 |
| 707.196_PM | -6.2 | 0.022 | 0.09 | -3.45 |
| 707.500_PA | -7.2 | 0.017 | 0.10 | -3.37 |
| 708.613_Cer | 7.7 | 0.016 | 20.74 | 4.37 |
| 714.591_HexCer/DGCC | -4.3 | 0.048 | 0.17 | -2.58 |
| 718.613_LPC | -4.3 | 0.048 | 0.14 | -2.83 |
| 719.463_PA | -5.7 | 0.027 | 0.15 | -2.72 |
| 721.457_PG | -5.5 | 0.028 | 0.17 | -2.54 |
| 721.480_PA | -4.7 | 0.037 | 0.13 | -2.97 |
| 728.607_DGCC | -8.0 | 0.015 | 0.05 | -4.19 |
| 729.484_PA | -8.5 | 0.014 | 0.13 | -2.97 |
| 730.622_HexCer | -5.6 | 0.027 | 0.19 | -2.43 |
| 731.499_PA/SM | -5.3 | 0.030 | 0.32 | -1.63 |
| 731.625_DG/TG | -5.4 | 0.028 | 0.14 | -2.81 |
| 732.345_PS | 5.7 | 0.026 | 6.84 | 2.77 |
| 732.503_PC | -9.4 | 0.012 | 0.24 | -2.09 |
| 733.516_PA | -5.9 | 0.025 | 0.12 | -3.01 |
| 742.287_PC | -8.3 | 0.014 | 0.03 | -4.91 |
| 745.479_PG | -4.8 | 0.036 | 0.13 | -2.97 |
| 747.473_SQDG | -4.9 | 0.035 | 0.35 | -1.51 |
| 756.590_PC | -5.2 | 0.031 | 0.14 | -2.81 |
| 758.519_PC/PE | -5.0 | 0.033 | 0.21 | -2.26 |
| 760.534_PC/PE | -4.9 | 0.035 | 0.32 | -1.64 |
| 762.540_PC/PE | -6.1 | 0.024 | 0.12 | -3.10 |
| 764.558_PC | -6.3 | 0.021 | 0.36 | -1.46 |
| 765.560_PC/PE | -7.2 | 0.017 | 0.23 | -2.10 |
| 770.570_PE/PC | -4.7 | 0.037 | 0.12 | -3.09 |
| 773.171_PM | -5.1 | 0.032 | 0.04 | -4.51 |
| 774.589_DGCC | -7.7 | 0.016 | 0.01 | -6.17 |
| 775.592_TG | -5.4 | 0.028 | 0.03 | -4.92 |
| 779.150_PM | -7.2 | 0.017 | 0.04 | -4.83 |
| 781.534_PG/TG | -4.9 | 0.035 | 0.26 | -1.95 |
| 782.546_DGCC | -8.2 | 0.015 | 0.28 | -1.86 |
| 789.561_PC | -4.5 | 0.043 | 0.29 | -1.80 |
| 802.536_PC | -4.6 | 0.038 | 0.13 | -2.91 |
| 823.631_PC | -9.3 | 0.012 | 0.04 | -4.52 |
| 824.637_PC | -16.5 | 0.012 | 0.03 | -5.25 |
| 825.648_PC | -6.2 | 0.022 | 0.05 | -4.26 |
| 826.651_PC | -5.9 | 0.025 | 0.03 | -4.99 |
| 831.270_PIP | -6.6 | 0.019 | 0.04 | -4.75 |
| 853.681_DG/TG | -10.7 | 0.012 | 0.04 | -4.68 |
| 871.717_TG | -4.8 | 0.035 | 0.17 | -2.58 |
| 913.664_TG | -5.3 | 0.029 | 0.14 | -2.81 |
| 914.667_TG | -6.7 | 0.018 | 0.07 | -3.93 |
| 939.680_TG | -7.4 | 0.017 | 0.09 | -3.50 |
| 953.835_TG | -5.0 | 0.033 | 0.10 | -3.37 |
| 967.711_TG | -6.9 | 0.017 | 0.10 | -3.38 |
| 969.315_PIP2 | -11.7 | 0.012 | 0.03 | -4.93 |
| 988.686_PC | -6.8 | 0.018 | 0.11 | -3.13 |
| 994.575_MIPC/Hex2Cer | -9.2 | 0.012 | 0.18 | -2.47 |
| 1009.57_PI | -5.8 | 0.026 | 0.24 | -2.07 |
| 1019.61_PIP/DGDG | -5.4 | 0.029 | 0.21 | -2.28 |
| 1035.29_PIP | -8.0 | 0.015 | 0.04 | -4.71 |
| 1041.28_PIP | -10.5 | 0.012 | 0.01 | -6.29 |
| 1050.7_MIPC/Hex2Cer | -8.0 | 0.015 | 0.05 | -4.39 |
| 1065.57_PIP/PIP2 | -7.2 | 0.017 | 0.11 | -3.17 |
| 1081.54_PIP/PIP2 | -5.9 | 0.025 | 0.16 | -2.65 |
| 1082.54_PIP/PIP2 | -6.3 | 0.021 | 0.23 | -2.13 |
| 1094.69_MIPC/Hex2Cer | -45.4 | 0.001 | 0.03 | -5.21 |
| 1108.71_MIPC/Hex2Cer | -9.4 | 0.012 | 0.05 | -4.39 |
| 1183.62_PIP2 | -6.1 | 0.024 | 0.09 | -3.42 |
| 356.195 | -13.8 | 0.012 | 0.003 | -8.57 |
| 395.096 | -9.4 | 0.012 | 0.01 | -6.64 |
| 396.797 | 6.2 | 0.023 | 37.61 | 5.23 |
| 408.191 | -7.1 | 0.017 | 0.03 | -4.90 |
| 453.184 | -5.5 | 0.027 | 0.03 | -5.25 |
| 548.256 | -5.1 | 0.032 | 0.09 | -3.48 |
| 569.26 | -11.9 | 0.012 | 0.02 | -5.41 |
| 584.128 | -5.4 | 0.028 | 0.06 | -4.04 |
| 585.525 | -7.0 | 0.017 | 0.05 | -4.31 |
| 591.243 | -8.1 | 0.015 | 0.10 | -3.39 |
| 612.519 | 4.3 | 0.047 | 7.61 | 2.93 |
| 678.567 | 7.5 | 0.016 | 10.44 | 3.38 |
| 683.601 | 6.9 | 0.017 | 17.17 | 4.10 |
| 744.638 | -9.2 | 0.012 | 0.05 | -4.36 |
| 745.457 | -4.7 | 0.036 | 0.14 | -2.79 |
| 756.503 | -4.9 | 0.035 | 0.37 | -1.44 |
| 779.478 | -10.2 | 0.012 | 0.01 | -6.24 |
| 779.539 | -5.0 | 0.033 | 0.24 | -2.03 |
| 786.277 | -7.5 | 0.016 | 0.02 | -5.51 |
| 787.282 | -9.3 | 0.012 | 0.03 | -4.91 |
| 797.617 | -11.8 | 0.012 | 0.04 | -4.64 |
| 811.129 | -6.0 | 0.025 | 0.08 | -3.71 |
| 817.68 | -5.6 | 0.027 | 0.13 | -2.98 |
| 821.625 | 7.8 | 0.016 | 5.10 | 2.35 |
| 830.268 | -4.5 | 0.041 | 0.05 | -4.35 |
| 850.235 | -4.9 | 0.035 | 0.09 | -3.55 |
| 854.235 | -5.5 | 0.027 | 0.04 | -4.68 |
| 875.235 | -7.0 | 0.017 | 0.04 | -4.77 |
| 876.524 | -7.0 | 0.017 | 0.14 | -2.82 |
| 877.527 | -4.8 | 0.036 | 0.17 | -2.59 |
| 878.816 | -9.3 | 0.012 | 0.08 | -3.56 |
| 880.833 | -9.4 | 0.012 | 0.05 | -4.35 |
| 893.503 | -5.8 | 0.026 | 0.35 | -1.53 |
| 896.238 | -7.1 | 0.017 | 0.04 | -4.70 |
| 902.817 | -10.9 | 0.012 | 0.05 | -4.43 |
| 903.357 | -11.9 | 0.012 | 0.03 | -5.14 |
| 905.651 | -7.5 | 0.016 | 0.07 | -3.81 |
| 906.653 | -10.0 | 0.012 | 0.05 | -4.42 |
| 906.847 | -8.4 | 0.014 | 0.07 | -3.84 |
| 907.85 | -8.4 | 0.014 | 0.09 | -3.44 |
| 912.212 | -6.9 | 0.017 | 0.07 | -3.74 |
| 926.818 | -7.4 | 0.017 | 0.05 | -4.32 |
| 927.632 | -11.6 | 0.012 | 0.04 | -4.58 |
| 928.833 | -8.0 | 0.015 | 0.05 | -4.33 |
| 929.836 | -7.1 | 0.017 | 0.05 | -4.42 |
| 930.849 | -8.1 | 0.015 | 0.06 | -4.06 |
| 932.866 | -6.9 | 0.017 | 0.13 | -2.98 |
| 952.832 | -5.2 | 0.031 | 0.11 | -3.13 |
| 953.599 | -4.8 | 0.035 | 0.18 | -2.46 |
| 954.603 | -4.5 | 0.041 | 0.18 | -2.47 |
| 967.577 | -6.2 | 0.022 | 0.14 | -2.85 |
| 968.58 | -5.2 | 0.030 | 0.13 | -3.00 |
| 969.572 | -4.8 | 0.035 | 0.20 | -2.29 |
| 969.594 | -5.2 | 0.030 | 0.13 | -2.90 |
| 970.599 | -5.7 | 0.026 | 0.12 | -3.06 |
| 993.574 | -4.3 | 0.048 | 0.18 | -2.44 |
| 993.592 | -6.9 | 0.017 | 0.17 | -2.59 |
| 994.598 | -6.4 | 0.021 | 0.18 | -2.50 |
| 1013.31 | -13.3 | 0.012 | 0.02 | -5.57 |
| 1164.36 | -11.7 | 0.012 | 0.04 | -4.79 |
| 1165.36 | -8.9 | 0.013 | 0.05 | -4.42 |
| 1168.66 | -4.7 | 0.037 | 0.33 | -1.61 |
| 1170.69 | -5.6 | 0.027 | 0.06 | -3.95 |
| 1182.62 | -5.5 | 0.027 | 0.09 | -3.48 |
| 1208.35 | -10.0 | 0.012 | 0.02 | -5.52 |
| 1209.36 | -11.2 | 0.012 | 0.03 | -5.17 |
| 1214.63 | -5.1 | 0.032 | 0.09 | -3.43 |

*A fold change (FC) > 1 indicate that the relative intensity of this metabolite is higher in B1-anemones, whereas a FC < 1 indicates that it is higher in C1-anemones. For instance, a FC value of 0.1 means that the relative intensity of this metabolite is 10 times higher in C1-anemones than B1-anemones.
