# Supplementary material for: Spatial metabolomics for symbiotic marine invertebrates
Source: Life Sci Alliance. 2023 May 18;6(8):e202301900. doi: 10.26508/lsa.202301900 (PMC10200813; doi:10.26508/lsa.202301900)
Supplement: Supplementary file 7 [file LSA-2023-01900_TableS6.docx]

**Table S6. Details of Symbiodiniaceae culture used to inoculate the anemones *E. diaphana*.**

| **Species** | **ITS2** | **Details** | **Culture ID** | **℃** | **Original host** | **Host origin** |
| --- | --- | --- | --- | --- | --- | --- |
| *Breviolum minutum* | B1 | Homologous,  wild-type | SCF 127-01 | 27 | *E. diaphana* | Central GBR, Australia |
| *Cladocopium* *proliferum* | C1 | Heterologous, wild-type | SCF 055-01.10 | 27 | *Acropora tenuis* | Magnetic Island, Australia |
